# Patellar tendon reconstruction using LARS ligament: surgical technique and case report

**DOI:** 10.1007/s11751-010-0101-0

**Published:** 2011-01-14

**Authors:** Soulat Naim, Nikolaos Gougoulias, David Griffiths

**Affiliations:** 1 University Hospital North Staffordshire, Stoke on Trent, UK; 2 Orthopaedic Department, City General, Newcastle Road, Stoke-on-Trent, ST4 6QG UK; 3 Frimley Park Hospital, Portsmouth Road, Frimley, Surrey, GU16 7UJ UK

**Keywords:** Patellar tendon, Rupture, LARS, Extensor mechanism, Reconstruction

## Abstract

Neglected patella tendon ruptures require reconstruction using tendon grafts. The LARS ligament has been successfully used in cruciate and collateral knee ligament reconstruction. We present a technique using LARS ligament for the reconstruction of a chronic patella tendon rupture in a low-demand patient. The result after 1-year follow-up was deemed successful.

## Introduction

Loss of function of the extensor mechanism may be a result of trauma, multiple operations or tumor resection and results in significant disability. If primary repair is not possible, reconstruction requires the use of tendon grafts [1]. Autologous tendon (e.g., hamstrings) grafting is a well-established technique and was described more than 50 years ago as a two-stage procedure [2]. More recent publications describe one-stage techniques using hamstrings tendons to reconstruct the extensor mechanism [3, 4]. In the elderly, however, the quality and strength of soft tissues are diminished and harvesting of tendons adds surgical morbidity. Furthermore, the prolonged rehabilitation required is not ideal for this patient population. Therefore, allografts [5, 6] and synthetic ligament grafts [7] can provide an alternative. The use of LARS ligament grafts (LARS Ligament, J. K. Orthomedic, Dollard-des-Ormeaux, Quebec, Canada) has been previously used for the reconstruction of isolated [8, 9] or multiple [10] knee ligament injuries and for reconstruction of the extensor mechanism after resection of malignant tumors around the knee [11].

This paper presents the surgical technique and clinical outcome at 1-year follow-up, of the reconstruction of a neglected, traumatic, and complete extensor mechanism deficiency in an elderly patient.

## Case report

A 79-year-old woman, with no significant past medical history, had a fall landing on her flexed left knee. As a consequence, she was unable to actively extend her knee and straight-leg raise. The patient walked with difficulty using a stick and did not seek medical attention until a year later. The patient was of short stature had a body mass index (BMI) of 31. There was gross quadriceps wasting, the patella tendon was not palpable, and the patella was proximally displaced. She had no active knee extension. Plain radiographs revealed a patella alta. An ultrasound scan confirmed complete deficiency of the patella tendon. It was agreed to reconstruct her tendon using synthetic ligaments.

**Fig. 1:**
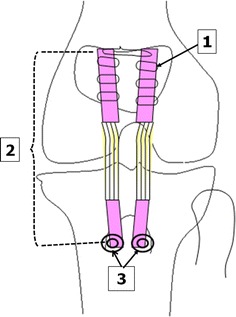
Illustration of surgical technique: Ethibond sutures [1] have been used (through fibrous tissue overlying the patella) to secure two LARS ligament grafts [2] to the patella. Interference screws [3] were used for tibial fixation

The operation was performed by the senior author (DG), with the use of a thigh tourniquet inflated to 300 mm Hg. Antibiotic prophylaxis was given. A midline skin incision was used. Findings were complete absence of the patella tendon and deficiency of the adjacent retinacular tissue. The remaining tissue was unsuitable for primary repair. Two bundles of LARS ligament were used. The rounded end was fixed to the tibial tuberosity with an interference screw (Fig. 1). The flattened end passed through tunnel prepared deep to the fibrous tissue covering the anterior aspect of the patella. Each bundle was secured with three Ethibond sutures, after the patella had been reduced to a satisfactory position. The repair and the adjacent retinacular tissue were reinforced with a Vicryl mesh. The wound was closed in layers. No drain was used. Operating time was 45 min.

The knee was splinted (cricket pad splint) in extension for 6 weeks. In the meantime, the patient was mobilizing as able with crutches and was encouraged to perform straight-leg raising exercises to strengthen the quadriceps muscle. Free mobilization and physiotherapist-guided knee flexion were initiated 6 weeks postoperatively. The patient was reviewed regularly in the outpatients’ clinic for 1 year.

**Fig. 2:**
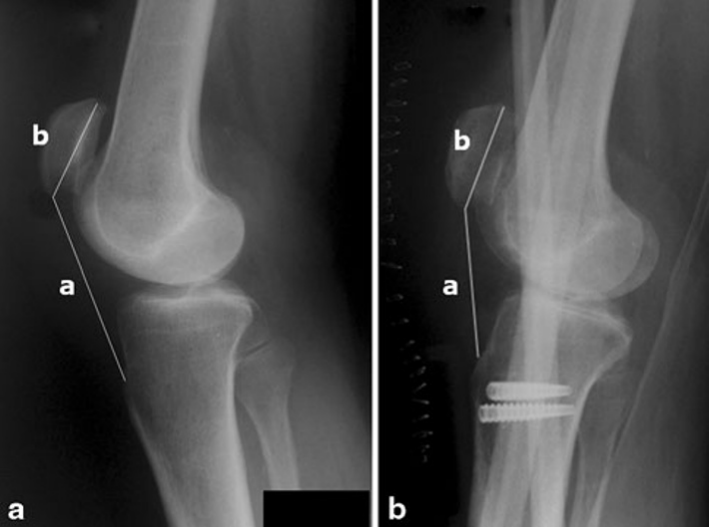
The Insall/Salvati ratio (a/b = length of patella tendon/to length of patella) improved from 1.9 preoperatively (**a**) to 1.3 immediately postoperatively (**b**)

**Fig. 3:**
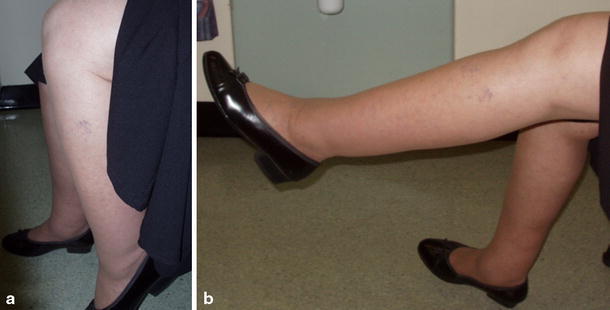
Follow-up at 1 year after surgery: Active knee flexion (**a**) and extension (**b**) were very satisfactory

The Insall/Salvati ratio [12] improved from 1.9 preoperatively to 1.3 postoperatively (Fig. 2a, b). The patient was able to perform active straight-leg raising as early as 2 weeks after her operation, when examined in the clinic. At 1-year follow-up, she was able to mobilize unrestricted, had regained full knee flexion (Fig. 3a), and had full power of extension (Fig. 3b).

## Discussion

Patella tendon rupture is a debilitating injury. Prompt diagnosis and treatment are essential to prevent retraction of the patella with subsequent adhesions and quadriceps contractures. In chronic ruptures, augmentation with tendon grafts is generally necessary [1]. Patients who undergo delayed repair are at risk for a compromised result secondary to loss of full knee flexion and decreased quadriceps strength, although a functional extensor mechanism is likely to be re-established [1]. The use of autologous hamstrings tendon grafts provides a viable option for successful reconstruction [3, 4]. Other authors have reported the use of Achilles tendon allografts, and the result was deemed successful [5, 6]. Artificial ligaments can be a useful tool in ligament reconstruction, avoiding the necessity of tendon-harvesting procedures and the possibility of donor site morbidity. Carbon fiber has been used to repair ruptures of the extensor mechanism of the knee involving either the patellar ligament or the quadriceps tendon, and the results in five patients were encouraging [7]. Extensor mechanism deficiencies following radical tumor resection around the knee have been previously reconstructed with the LARS ligament revealing 59% good/excellent results [11].

The use of the LARS ligament is well established in cases of cruciate and collateral knee ligament reconstruction [8–10]. Advantages of the use of the LARS ligament are as follows: (1) avoidance of donor site morbidity, (2) the ligaments’ mechanical properties, allowing early mobilization and quicker rehabilitation, (3) no evidence of tissue intolerance to the artificial material, (4) allowance of fibroblast ingrowth around the artificial ligament bundles [13], and (5) the possibility of repeating the reconstruction in case of failure. Furthermore, additional cerclage wire stabilization that requires removal [3, 5] is avoided by the presented technique.

For these reasons, we used LARS ligament for the reconstruction of a neglected complete patella tendon deficiency in an elderly, low-demand patient, with a successful 1-year follow-up. The surgical technique presented in the current paper is simple and reproducible.
